# The pilot and evaluation of a postnatal support group for Iraqi women in the year following the birth of their baby

**DOI:** 10.3389/fpsyg.2014.00016

**Published:** 2014-01-30

**Authors:** Rosanna M. Rooney, Robert T. Kane, Bernadette Wright, Vanessa Gent, Taralisa Di Ciano, Vincent Mancini

**Affiliations:** School of Psychology and Speech Pathology, Curtin University, PerthWA, Australia

**Keywords:** postnatal, Iraqi, cross-cultural, support group, postnatal depression, Iraqi women

## Abstract

The current study involved conducting a pilot test of a culturally sensitive support group program developed to assist Iraqi women in the year following the birth of their baby (CSSG-B) in Perth, Western Australia. The aim of this study was to evaluate the social validity of the program. It was hypothesized that women involved in the program would find the program to be socially valid and culturally appropriate, and will also report lower levels of depressive symptomatology and higher levels of social support, following the group intervention. Participants were 12 Iraqi Arabic speaking women, who had a child less than 12 months of age. The program was based on Iraqi women's explanatory models (Kleinman, 1978; Di Ciano et al., 2010) of the birth and motherhood experience. Social validity ratings were obtained during the implementation of the program in order to assess the level of acceptability of the intervention. A one-group pre-test–post-test design was used to determine if depressive symptoms had decreased during the course of the intervention and social support had increased. Results indicated that Iraqi Arabic speaking women found the support group intervention acceptable and relevant and there was a significant decrease in scores on the Edinburgh Postnatal Depression Scale (EPDS) from pre-test to post-test. These results that the culturally sensitive group intervention was culturally acceptable and was associated with decreased levels of depressive symptomatology.

## Introduction

Almost a quarter of Australia's resident population is now born overseas, with the most rapid growth of migrants coming from nations such as Burma, Sudan, Afghanistan, and Iraq (Australian Bureau of Statistics, 2001). In Perth, Western Australia, there was a 163% increase of Iraqi people migrating to Perth between 1996 and 2001 (Australian Bureau of Statistics, 2001; Office of Multicultural Interests, 2001). Migration may signify a marked change in the individual's circumstances, including loss of income, qualifications, status, prejudice, and discrimination (National Mental Health Strategy, 2003; Rooney et al., 2012). These increased stressors may be compounded by traumatic events experienced prior to migration and the isolation from family, religious, and cultural support networks. Such factors have been identified as placing people from culturally and linguistically diverse (CALD) communities at an increased risk of developing psychological disorders, including postnatal depression (Minas et al., 1996; National Mental Health Strategy, 2003).

Postnatal depression (PND) is a phenomenon that affects women from a wide range of cultures and is often characterized by irritability, low energy levels, anger, decreased interest in everyday activities and feelings of sadness and guilt over a period of at least 2 weeks (Pope et al., 1999; American Psychiatric Association, 2000; Craig et al., 2005; Fonte and Horton-Deutsch, 2005; Green et al., 2006). Although there is some symptomatic variation, PND transcends cultural boundaries, and there is growing concern regarding the impact of PND across cultures as the long term consequences may include impaired maternal-infant interactions, poor cognitive, emotional, and social outcomes for the children, marital disharmony and an increased likelihood of future episodes of depression (Pope et al., 1999; Murray et al., 2003; Boath et al., 2004; Poobalan et al., 2007; Korhonen et al., 2012).

PND occurs in approximately 15% of the general population in the first 12 months following childbirth (Pope et al., 1999; Asten et al., 2004; Boath et al., 2004). These rates have been estimated to be slightly higher in Arabic populations at 18–25%, although systematic prevalence studies on CALD populations are yet to be carried out (Ghubash and Abou-Saleh, 1997; Barnett et al., 1999; Chaaya et al., 2002; Small et al., 2003). While several risk factors for PND are universal (e.g., genetic predispositions, history of depression, social isolation, and new born temperament; Morse, 1993; Kaplan and Saddock, 1998; Nicholson, 1998; Milgrom et al., 2008), CALD individuals are exposed to additional risk factors. These factors include poor relationships with the husband and or extended family, multiparity, health concerns for the baby, not living with the extended family and unplanned pregnancy (Ghubash and Abou-Saleh, 1997; Danaci et al., 2002; Fonte and Horton-Deutsch, 2005).

The role of social support in the formation and maintenance of PND is an important factor to consider, particularly with relation to CALD women. Williams and Carmichael (1985) studied CALD mothers in Australia who were from multi-ethnic backgrounds and found those with few social supports had significantly higher rates of depression, whereas strong support networks including involvement of the father appeared to prevent depression. Similarly, a recent review undertaken by Barnett et al. (2005) examining PND in Vietnamese and Arabic women identified lack of emotional and practical support, including an inability to source assistance in attending to traditional postnatal rituals, as a risk factor for PND. Such findings express a need for further interventions that act to facilitate the development of additional social support as to buffer the risk of PND.

The diversity of CALD populations presents additional methodological issues that need to be addressed when reviewing the current state of research regarding PND in CALD populations. Symptoms of PND can vary across cultures, and this may include differences in the terminology, expression of symptoms and understanding of the disorder (Moon-Park and Dimigen, 1995; Bashiri and Spielvogel, 1999; Oates et al., 2004). Prominent PND assessment tools such as the Edinburgh Postnatal Depression Scale (EPDS; Cox et al., 1987), and measures of social support have been developed and validated in Western countries, using predominantly Western populations to validate these measures. Consequently, the validity of such measures may potentially be jeopardized as a result of a lack of cultural sensitivity to the way in which PND is characterized in CALD populations. To overcome this issue of a lack of cultural sensitivity, the developers of the EPDS and other researchers have validated the EPDS in a wide range of cultures including Taiwanese, Japanese, Moroccan, Turkish, and Arabic (Danaci et al., 2002; Agoub et al., 2005; Teng et al., 2005). With specific reference to Arabic women, researchers have made the recommendation of lower, culturally sensitive cut-off scores of 9–10 amongst Arabic women compared to the usual cut-off of 13 (Kleinman, 1978; Cox et al., 1987; Ghubash et al., 1997; Seah et al., 2002; Matthey et al., 2006).

While a small body of research has examined the utilization and preference of treatments for PND amongst different cultural groups, few studies have evaluated treatment outcomes for CALD women. In a qualitative study, Nahas et al. (1999) found it was beneficial for Arabic women to attend centers with other Arabic women where they could assemble as a group and discuss their concerns and life in Australia. Barnett et al. (2005) examined the uptake of psychosocial interventions to prevent PND amongst postnatal Vietnamese and Arabic women. Participants were offered a choice of face-to-face counseling, telephone support, antenatal classes, home visiting, or group therapy. Phone support was the preferred option for both ethnic groups, with the Arabic women preferring antenatal classes and home visits to individual counseling, in which decreases in PND were reported. However, a shortcoming of the study was the inability to distinguish which intervention has the greatest impact on reducing PND symptoms. In addition, neither of these studies examined which specific components of each intervention the CALD women participants found particularly beneficial, nor were the interventions specifically tailored to CALD populations; the interventions were not culturally specific, nor was an explanatory model used to determine different cultural perspectives of PND.

Explanatory models offer a cultural explanation of how health and illness is constructed, and emphasizes the importance of understanding individual cultural health care beliefs, and the potential influence that culture has on health (Kleinman, 1978). Failure to recognize and incorporate explanatory models when working with CALD communities can result in misinterpretation, failure of the client to engage in services, and poor treatment outcomes (Rooney et al., 1997, 2006; Bhui and Bhugra, 2002). Two recent studies have incorporated an explanatory model in order to develop interventions for postnatal depression that are culturally appropriate.

Down et al. (2005) examined the antenatal and postnatal experience of Ethiopian, Sudanese and Iraqi women who had given birth 1–2 years previously and had immigrated to Australia. Using focus groups run by women from the same cultural and language background they found women from each respective cultural group reported that they suffered isolation, loneliness, confusion, fear, and high levels of anxiety in the antenatal period, which the women felt was exacerbated by poor social supports. The women also reported anxiety related to their inability to uphold traditional birthing rituals and practices involving their mothers and extended family. The majority of women expressed a desire to take part in a social support group, facilitated by someone from within their community to assist them with support and advice on mothering, traditional cultural practices and to discuss issues pertinent to their cultural background and current situation.

Di Ciano et al. (2010) conducted in depth interviews with Iraqi Arabic speaking mothers living in Australia, with the aim of understanding their explanatory models surrounding motherhood and migration in Australia. Participants were found to be reluctant to express their need for practical and emotional support to their husbands and friends, as it was important for them to appear to be coping. The women indicated they would be willing to attend a culturally appropriate postpartum support group from which they could access information and interact with other mothers encountering similar issues and concerns. Topics they felt were important to discuss included stress, depression and parenting in a western society with a lack of family support. Provision of transport, interpreters, and childcare facilities were identified as crucial to facilitate attendance. Trust, group confidentiality, and rapport with the facilitators were considered salient factors in the success of any intervention. These two studies exemplify the importance of social validity as an important factor to consider when developing, implementing, and evaluating PND interventions in CALD populations.

Social validity refers to the social judgment of the relevance and acceptability of behavioral interventions (Kazdin, 1977; Wolf, 1978). One way of assessing whether an intervention is culturally acceptable and viable to a community is through an examination of the social validity of the intervention. The success of any psychological intervention is dependent on the acceptability and relevance of the intervention to the particular target group. This is likely to be even more salient amongst groups from diverse cultural backgrounds where there has been a reluctance to engage in mainstream interventions (NSW Review, 1994, 1996; Rooney et al., 1997; Taouk, 2004). Subsequently, an evaluation of social validity is an important component of developing effective programs and intervention strategies.

No research to date involving postnatal depression interventions has incorporated women's explanatory models and evaluated the program's ability to prevent or reduce PND in CALD women. The current study builds on the research undertaken by Down et al. (2005) and Di Ciano et al. (2010) in Western Australia in which CALD women from Iraqi, Sudanese and Ethiopian groups requested the implementation of a culturally sensitive intervention in order to access information and support during the post-partum period, specifically focusing on Iraqi women.

The current study involved conducting a pilot test of a culturally sensitive support group program developed to assist Iraqi women in the year following the birth of their baby (CSSG-B), in Perth, Western Australia. The aim of this study was to evaluate the social validity of the program. As the program is based on Iraqi women's explanatory models of motherhood and PND, it is hypothesized that the CSSG-B intervention will be socially validated by the CALD Iraqi women involved in the intervention. In addition, it is also hypothesized that participants involved in this pilot study will have lower levels of PND symptomology compared to their pre-test scores, and higher levels of social support following completion of the intervention. As the present study is a pilot study, no control group was employed. As such, the pre-post-test changes on the outcome variables may reflect the influence of extraneous variables rather than the intervention itself. However, the demonstration of within group pre-post changes is first required in order to justify conducting a more resource-intensive randomized control trial.

## Methods

### Participants

Twelve Iraqi women participated in the present study. All participants spoke Arabic as a first language, and had a child born in Australia aged between 6-weeks and 1 year of age at the time of referral. Demographic information for participants are presented in Table 1.

**Table 1:** **Participant demographics**.

Group numbers	12	
Age range	24–43 years	*M* = 32
Marital status	10 married, 2 divorced	
Religion	All Muslim	
Number of children	2–6	*M* = 4
Children age range	5 months–19 years	
Educational level	1 no schooling	
(In country of origin)	3 primary, 6 secondary, 2 further education	
Previous occupation	6 home duties	
	3 students	
	1 tailor	
	2 teachers	
Occupation in Australia	11 home duties, 1 student	
English literacy	8 ISLPRS Level 1	
	3 ISLPRS Level 2	
	1 ISLPRS Level 3	
Years in Australia	3–9 years	*M* = 5
Child been in crèche before	3 yes, 9 no	

*M, mean; ISLPRS, International Second Language Proficiency Rating Scale as assessed by Adult Migrant English Programme; Level 2 is the minimum language proficiency required for basic functioning in a social or employment setting*.

A total of 16 participants took part in the final session, as the initial participants requested to bring other women to the group. However, only the data of participants who attended a minimum of five of the six sessions were included for analysis.

### Measures

#### Social validity measure

Two measures of social validity were implemented in the present study.

#### The participant process evaluation form

In order to examine how participants evaluated the program, an adapted Student Process Evaluation Form was used. Originally used in a selected trial of the Penn Prevention Program (Roberts et al., 2003), this measure was adapted to suit the present study. This form contains three distinct sections:

***Section A: Session evaluations***. Participants were provided with the opportunity to indicate which components of the session they enjoyed the most and the least on a weekly basis. They were provided with a 5-point Likert scale ranging from 1 *(not at all enjoyable*) to 5 *(I enjoyed it very much)*.

***Section B: Overall program evaluation***. Participants responded to a series of 15 different statements to indicate their satisfaction with the program as a whole on a 5-point Likert scale ranging from 1 (*not at all)* to 5 (*very much*).

***Section C: Qualitative program evaluation***. Five open-ended statements gave the participants the opportunity to describe components of the program they enjoyed the most and the least and which information they utilized most often.

#### Facilitator logbook

Facilitators completed a logbook throughout the duration of the program. These logbooks contained checklists and rating scales to assess the quality of how each session's content was delivered. Five 10-point rating scales were provided for facilitators to rate the quality of their presentation in terms of overall success of the session, their preparation, presentation, rapport with the group and the level of cohesion and support within the group. Independent observers also completed identical checklists.

#### Intervention outcome measures

Two different measures were employed in order to examine participant outcomes relating to postnatal depression and social support.

#### The edinburgh postnatal depression scale (EPDs)

The EPDS (Cox et al., 1987) is a 10-item self-report measure, developed specifically to screen women for PND. The scale reports high levels of internal reliability (α = 0.88) and a split half reliability of 0.84 as a screening tool in English speaking women (Cox et al., 1987). Translated versions of the EPDS have been used as a cross-cultural measure of PND. Specific studies that have examined the validity of the EPDS translated into Arabic have found the scale to report similarly high levels of internal reliability (α = 0.84) as the English version. Lower cut-off scores of 9–10 have been used with women completing the Arabic version of the EPDS to increase the sensitivity of the scale (Ghubash et al., 1997). For this reason, the present study used a cut-off score of 10 to assess PND.

#### The norbeck social support questionnaire (NSSQ)

The outcome measure used to assess social support and support networks was the NSSQ (Norbeck et al., 1983). The NSSQ is an 8-item measure that first asks participants that asks participants to rate the quality and degree of support they receive from each person they list as a significant person in their life. The NSSQ has moderate levels of concurrent validity and high internal consistency reliability (range 0.89–0.97), with strong correlations between affect and affirmation subscales (range 0.95–0.98) and between aid and affect and affirmation [range 0.72–0.78; (Norbeck et al., 1983)].

### Procedure

Two female, Arabic and English speaking bilingual workers were recruited to assist in the delivery of session content of the current study. These workers received additional, specialized training from the principal researcher in order to ensure preparedness. This training addressed topics relating to the signs and symptoms of PND amongst CALD women, the administration of the EPDS and the NSSQ, follow-up for women with high EPDS scores, cultural sensitivity, potential ethical issues, and also debriefing of participants. These bilingual workers were actively involved in the development and refinement of the questions that were used to assess the social validity of the program, providing recommendations and adjustments to items in order to make sure they were culturally appropriate. All of the measures used in the present study were translated into Arabic, and then back translated into English by professionals from the National Accredited Authority for Translators and Interpreters.

Participants were recruited through convenience sampling, using community based child health nurses. Those who gave their written or verbal consent to participate in the interviews were then phoned by the bilingual workers, who then briefed the prospective participants on the details of the study, and arranged an interview with participants in their own home. During this visit, participants completed the EPDS and the NSSQ. Participants who required transport or access to childcare facilities during the time of the interview were identified during the phone conversation, and were provided.

#### Facilitators

Four facilitators were actively involved over the duration of the intervention. These facilitators included a Child Health Nurse, and Psychologist, and two women from the local Arabic community. Each facilitator had previous experience conducting similar groups, and was aware of the time and content limitations. Each was also experienced in working with CALD groups and experienced in cross-cultural awareness. Facilitators were encouraged to promote engagement the active participation and contribution of participants in such a way that facilitated the development of support, rather than just dissemination of information. The sessions were co-facilitated by a Clinical Psychology Master's student, and also a bilingual worker. An Iraqi interpreter was also present at each session. At the end of each session, facilitators reported on the integrity of the content of the session by using checklists. The independent observers, who were clinical psychology masters students, also completed these same checklists.

#### Session content

Previous research by Di Ciano et al. (2010) incorporated in depth interviews with seven Iraqi women with postpartum depression to understand what they felt were important to incorporate in a culturally sensitive postnatal support group intervention. This information was then taken to a reference group that consisted of Arabic women, clinical psychologists cross cultural health professionals, child health nurses, and representatives from Islamic women's organizations. The information provided by the women and the reference group guided the content of the sessions and was used to form an intervention manual.

The themes of the six-session intervention are outlined in Table 2. The sessions were held on the same day of the week at a time the participants had identified as being the most convenient. The sessions ran for 2 h duration and included morning tea. Lunch was held at the third session, as this was a celebration of Iraqi culture and sharing of food. At the end of each session, participants completed a rating scale to indicate how enjoyable and useful they found the session. Following the completion of the program, research assistants administered program evaluation forms to the whole group. A final reunion meeting and follow-up session took place approximately 6 weeks after the final session. At the participants' request, this was a picnic. At this session, the participants were encouraged to explore ways of continuing to provide support for one another and to remain in contact.

**Table 2:** **Session content of the Iraqi support group**.

**Session 1**	**Session 2**
Confidentiality and rules	Breastfeeding
The birthing experience	Cultural issues and beliefs
Expectations of motherhood	
**Session 3**	**Session 4**
Foods benefiting pregnant women and after childbirth	Challenges to traditional role of wife/mother
Iraqi food/culture in Australia	Discipline issues with children
Sharing of food	Parenting without family support
	Maintaining culture and traditions
**Session 5**	**Session 6**
Stress and depression following childbirth	Services in the community
Common signs of stress and depression	Psychological services
Causes of depression	Multicultural groups
Fear and stigma	Access issues/staying in touch
How to deal with stress and depression	
**Session 7**	
Reunion picnic	

## Results

### Data screening and assumption testing

For each quantitative analysis, data were screened for violation of the relevant assumptions using guidelines provided by Coakes and Steed (2003). Data were only collected from participants who were present for the entire group session. Outliers were identified as scores that fell more than 3.3 standard deviations from the mean and were disconnected from the remainder of the scores (Tabachnick and Fidell, 2001).

### Global participant evaluations

Participants rated the overall program at the end of the final session. The results of these analyses are displayed in Table 3.

**Table 3:** **Mean participant ratings of the enjoyment of the overall program (1, not at all enjoyable; 5, I enjoyed it very much), with the percentage of participants who gave ratings of 4 and above**.

**Item**	**Mean**	***SD***	**%>4**
I look forward to the sessions each week	5.00	0.00	100.0
The sessions were easy to understand	4.80	0.42	100.0
The program was useful in my everyday life	4.60	0.70	90.00
The program helped me to have a better understanding of the supports available to me	4.90	0.32	100.0
The program helped me to get along better with my baby/children	4.00	0.47	90.00
The session seemed appropriate to my cultural needs and background	4.70	0.48	100.0
The program helped me to feel more confident as a mother	4.80	0.42	100.0
The program helped me feel more connected with women from my own community	4.70	0.48	100.0
The program has helped me to cope with stress in my everyday life	4.00	0.67	80.00
The program has helped me to feel happier about everyday life	4.10	0.88	70.00
The program has helped me to get to know other women with young babies	4.60	0.70	90.00
I shared information I learnt in the program with my husband and family	4.70	0.48	100.0
My husband and family has been supportive of my involvement in the program	4.90	0.31	100.0
I feel I have increased levels of support in my life	4.50	0.52	100.0
I would recommend the program to other women with a baby	5.00	0.00	100.0

Eleven of the 12 participants gave mean ratings of four and above for the overall group ratings on a scale of 1–5. A detailed account of the open-ended statements for each question is provided below:

***The sessions I enjoyed the most***. Participants reported the sessions that incorporated discussion on overcoming postnatal depression and the psychological state of pregnancy as well as having opportunities to discuss their feelings were beneficial. For example, one participant indicated, “The sessions about the differences in the support that were available in Iraq in comparison to what is available here gave me a chance to express my feelings.”

***The sessions I did not enjoy***. All participants stated they enjoyed the sessions. Common statements included “I enjoyed all of the sessions I took part in.”

***The information I use most from the program***. Many participants indicated the sessions that allowed them to discuss parenting and child raising concerns were useful. Common statements included “Listening to the information that teaches us how to raise children.” Some participants indicated that they worried about their families that were in Iraq and being able to discuss this with other women helped to alleviate this concern. One respondent remarked, “Discussing the worry I felt about not seeing my brother was helpful.”

***The program could be improved by***. Many participants reported the program was helpful as it was. Suggestions to improve the program included “Adding new and more variable topics,” and “Doubling the length of the program to twice per week or more.”

***What other areas should we include in the program?*** Several participants felt English classes should be incorporated into the program. Others expressed a desire to engage in activities that were more social, with one participant reporting that “It would be good to have more activities outside the center during the program.” Some participants suggested ongoing support especially about parenting concerns would be beneficial “Talking about the future more and how we can treat problems that relate to the children.”

***Individual session ratings***. At the end of each session the participants were required to rate each session objective in terms of their enjoyment on a 1–5 scale. The summary statistics for these ratings are provided in Table 4. Ten of the 12 participants gave ratings of four and above for the session objectives.

**Table 4:** **Mean participant ratings of the enjoyment of the individual Sessions (1, not at all enjoyable; 5, I enjoyed it very much), with the percentage of participants who gave ratings of 4 and above**.

**Content rated**	**Mean rating**	***SD***	**%>4**
**SESSION 1**			
Confidentiality and rules	5.00	0.00	100.0
The birthing experience	3.57	0.98	57.20
Expectations of motherhood	4.14	1.07	57.10
**SESSION 2**			
Breastfeeding	4.00	1.06	75.00
Cultural issues and beliefs	4.88	0.35	100.0
**SESSION 3**			
Foods benefiting pregnant women and after childbirth	4.38	0.52	100.0
Iraqi food and culture in Australia	5.00	0.00	100.0
Sharing of food	4.63	0.52	100.0
**SESSION 4**			
Challenges traditional wife/mother without family	4.14	0.90	71.50
Parenting without family assistance	4.14	1.06	57.10
Discipline issues and importance of communication	4.86	0.38	100.0
Maintaining cultural and religious traditions in a different culture.	4.00	1.00	57.20
Fears of unknown/fears of failure	3.58	0.53	57.10
**SESSION 5**			
Stress and depression following childbirth	4.71	0.49	100.0
Common signs of stress and depression	4.28	0.49	100.0
Causes of depression	4.33	0.52	100.0
Fear and stigma	3.67	0.82	100.0
How to deal with stress and depression	4.00	0.64	50.0
**SESSION 6**			
Services that are available in the community	4.78	0.44	100.0
Psychological services	4.67	0.71	88.90
Multicultural women's groups	5.00	0.00	100.0

***Program integrity and attendance***. Session checklists completed by the facilitators indicated that it was difficult to cover all of the session objectives in the allocated session time. In Sessions 1 and 2, there was an omission of two objectives. Session 4, 5, and 6 covered all content; however, facilitators across all sessions indicated they ran out of time before they were able to reiterate the key messages from the session. The independent observers and facilitators assessed the sessions on three different rating scales. The results of the observers' ratings and the facilitators' self-report ratings indicated there was 100% agreement on the content covered in each session. Both the independent observers and the facilitators were asked to rate the participants' satisfaction with each session objective as a score out of 10, with 1 = not satisfied, 5 = moderately satisfied and 10 = very satisfied. Overall the mean satisfaction ratings for observers was 7.77 (*SD* = 1.14) and facilitators 7.61 (*SD* = 1.34). A related samples *t*-test failed to find a difference in satisfaction ratings between the observers' and facilitators' ratings (*p* > 0.05).

***Facilitator and observer ratings***. The results of the facilitator's ratings and independent observer's ratings on the session presentation indicated that both the observers and the facilitators rated the quality of the sessions as high in terms of the overall success of the sessions, preparation, presentation, rapport with the group and the cohesion of the group. Mean observer and facilitator ratings were assessed using a 10-point scale where 1 = unacceptable, 5 = passable, and 10 = excellent. These scores were then compared using a related samples *t*-test. The results of these analyses are displayed in Table 5.

**Table 5:** **Mean observers and facilitators ratings for five rating scales on a scale of (1–10)**.

**Session aspect**	**Facilitator**	**Independent observer**	
	**Mean (*SD*)**	**Mean (*SD*)**	***t*-test (ns)**
Overall success of session	8.50 (1.04)	8.83 (0.41)	*t*_(−0.791)_ = 0.47
Preparation of session	8.00 (1.41)	8.50 (1.04)	*t*_(−0.889)_ = 0.42
Presentation of session	7.33 (1.75)	8.16 (0.75)	*t*_(−1.27)_ = 0.26
Rapport with the group	8.00 (1.67)	8.50 (1.04)	*t*_(−0.1.45)_ = 0.20
Group cohesion and support	9.16 (0.40)	9.00 (0.00)	*t*_(1.00)_ = 36

Attendance checklists indicated that five of participants had full attendance at all sessions, while three of participants missed up to two of the sessions. Attendance rates at each session did not fall below 70% (at least eight participants for each session). A follow-up picnic was held 6 weeks post-intervention. There was an increase in participants, with group numbers increasing from 12 to 16 for this session.

***Post-intervention changes in postnatal depression***. A one-factor within-subjects ANOVA was conducted to determine if participants' scores on the EPDS decreased from pre-test to post-test and from post-test to follow-up. There was a significant main effect for Time [*F*_(2, 22)_ = 9.86, *p* = 0.001, η^2^ = 0.473] indicating a significant decrease in EPDS scores across time (*Mean* EPDS pre-test = 12.024, *Mean* EPDS post-test = 8.17, *Mean* EPDS follow up = 7.905). *Post-hoc* tests of within-subjects contrasts showed a significant difference between pre-test and post-test [*F*_(1, 11)_ = 16.62, *p* = 0.002, η^2^ = 0.602], but no significant difference between post-test and follow-up [*F*_(1, 11)_ = 0.08, *p* = 0.780, η^2^ = 0.007].

Due to a return rate of only 10% on the Norbeck Social Support Questionnaire (NSSQ), analysis of results of differences between pre and post-test scores was not possible. The low return rates of the NSSQ were attributed to the duration and difficulty associated with completing the measure, despite its translation into Arabic. Inspection of pre-test responses revealed that seven of the participants relied solely on their husbands and daughters for support. Three participants reported relying on extended family and husbands, and two participants listed friends as a source of support.

## Discussion

The current study involved pilot testing and evaluating the social validity of a support group for postpartum Iraqi women. The aim of this study was to evaluate the social validity of the program Two major areas of social validity examined. First, the global social validity of the program, including participation rates, integrity of the program implementation and the participants' social validity ratings of the program and second, Participants' ratings of their enjoyment and the utility of the skills taught in each weekly session. The second aim of the current study was to explore the potential for the intervention to reduce PND symptoms and increase social supports for Iraqi mothers in the year following the birth of their baby. It was hypothesized that the post-test EPDS scores would be lower following the intervention when compared with pre-test EPDS scores and post-test levels of social support would have increased following the intervention when compared with pre-test levels. As the measure of social support (the NSSQ) had only a 10% return rate, results relating to social support could not be interpreted meaningfully.

### Global social validity

Initial consent to participate in a program has been suggested as an indicator of a program's global social validity (Foster and Mash, 1999). Initial attempts by child health nurses to recruit women directly into the intervention were unsuccessful. The use of an Iraqi bi-lingual worker resulted in an initial recruitment rate of 76%. This is in keeping with findings by Rooney et al. (2006) who examined clients preferred practitioners from their own cultural background, predominantly to overcome the language barriers. The high attendance rates of 70%, the increase in the number of participants following the recommendation of the program by the initial participants to other Iraqi women are indicators of the global acceptability of the treatment amongst this cultural group. In comparison, attrition rates of 63, 17, and 55% were found in other group interventions for PND (Brugha et al., 2000; Milgrom et al., 2005; Reay et al., 2005). The current results support Foster and Mash (1999) dimensions of treatment acceptability, in which low dropout rates, high rates of attendance and the ease of dissemination of the intervention can be used as consumer ratings of global treatment acceptability. This also highlights the importance of using an explanatory model in which the women's cultural needs were taken into consideration when planning the intervention, making the intervention culturally acceptable to them.

The results of the facilitators' ratings and independent observers' ratings on the session presentation indicated that both observers and facilitators rated the quality of the sessions as high in terms of the overall success of the sessions, the preparation, presentation, and rapport with the group. The results demonstrated that high levels of program fidelity could be obtained when sessions are delivered by culturally sensitive facilitators who are prepared to incorporate participants' explanatory models into the program. These findings are supported by numerous other studies that have indicated the need for culturally sensitive and collaborative interventions (McCarthy and Barnett, 1996; Nahas and Amasheh, 1999; Down et al., 2005; Di Ciano et al., 2010).

Participant responses regarding the overall global social validity indicated there was a high level of satisfaction with the intervention. All of the participants commented they would recommend the group to other woman with young babies and all of the women looked forward to the sessions each week. Results of the qualitative data suggested there were minimal changes recommended by the participants regarding the intervention. However, participants disclosed a desire for longer with English classes incorporated, and more opportunities to socialize outside of the center during the course of the program. Results support findings that indicated women from non-English speaking backgrounds may be more socially isolated as a result of language barriers (Williams and Carmichael, 1985; Barnett et al., 2005). However, findings also highlight the desire of these women to overcome these barriers, and suggest an inability to access services on their own accord. This factor has been highlighted previously as a barrier for effective service provision for CALD communities (McCarthy and Barnett, 1996).

### Participants' ratings of their enjoyment and utility of skills taught

The majority of the participants indicated they found all of the sessions enjoyable. The findings are consistent with research findings by Di Ciano et al. (2010) which suggested that Iraqi Arabic speaking mothers were receptive to a group in which they could discuss their postpartum health concerns and difficulties including depression and parenting in a western society. These results also suggest that disseminating information through groups may be a more effective way of addressing the low use of services such as child health clinics and external services (McCarthy and Barnett, 1996; Postmontier and Horowitz, 2004; Di Ciano et al., 2010). The participants indicated they found the sessions on parenting in a multicultural society the most useful, reporting they would be the most highly utilized of all the modules. These results are consistent with researchers who found that many mothers who have immigrated feel insecure in their ability to parent and maintain traditional cultural and role expectations (Nahas and Amasheh, 1999; Down et al., 2005; Di Ciano et al., 2010). It is plausible that this session may have been more salient to the women as the bilingual worker was able to recognize the traditional role expectations that remain and was able to work within the appropriate cultural guidelines as has been suggested by previous research (Di Ciano et al., 2010).

### Levels of depressive symptomatology

A significant decrease in EPDS scores from pre-test to post-test was found following the intervention, a decrease that was maintained at the 6-week follow up. This significant decrease in EPDS symptoms suggests that the intervention may have been instrumental in reducing symptoms of postnatal depression, however, as this was a pilot study and there was no control group, it is impossible to determine if the intervention led to a decrease in symptomatology or if it is a consequence of other extraneous variables. It does support results of the qualitative data taken from the women at the completion of the intervention where several participants stressed the value and benefit of being able to talk about the signs of symptoms of postnatal depression and stress. This is further supported by other findings amongst non-CALD populations where psychological treatments that included psychoeducation about PND were found to decrease raised EPDS scores in the first 6 weeks following an intervention (Scott and Freeman, 1992; Chabrol et al., 2002).

It was also revealed through the course of the study that women with symptoms of postnatal depression were reluctant to seek treatment. Only one participant who was referred for extra support out of four agreed to individual counseling and this was largely in the context of her physical health problems. Interestingly all the participants with elevated EPDS scores continued with the group intervention. This is consistent with findings that women from CALD communities are reluctant to engage in individual therapy due to the stigma associated with mental illness (Rooney et al., 2006). This supports recommendations by Spence et al. (2003) that universal treatment programs offered to all individuals within a community help prevent stigma and decrease the labeling effects of an individual being singled-out for treatment.

### Social support

The response rate was too low to enable carrying out the evaluation of the differences between levels of the NSSQ from pre-test to post-test. The low response rate of the NSSQ may have been due to the large size of this assessment inventory making it difficult to comprehend and time consuming to complete. A further explanation for the low return rate may be the cultural inappropriateness of administration of inventories that explore one's interpersonal supports and social connectedness. Inspection of pre-test responses indicated participants relied heavily on their husbands and children for emotional and tangible support and received very little support from outside sources. This is consistent with previous research which has found that the cultural beliefs of Arabic women may prevent them from actively engaging in seeking practical or emotional support from friends and at times their husbands, preferring to be seen as coping and not wanting to impose on others (Nahas and Amasheh, 1999). These findings also suggest a social support group may assist women from CALD backgrounds to access support within their own cultural framework.

### Limitations of the study

The results of this pilot study should be treated with caution because of the small sample size, which included only 12 participants and lack of a control group. Without a control group, it is difficult to conclude that the significant reduction in PND symptoms were due to the group or other factors. Other limitations included the effects of social desirability of responses. It is possible the participants filled in the questionnaires in the way they felt the researcher desired. An attempt was made to overcome this by getting the participants to complete the questionnaires at home, however, low return rates indicated the need for completion prior to leaving the session. In addition, the use of a large questionnaire such as the NSSQ may have been culturally irrelevant to the participants, resulting in a low return rate. This remains an ongoing difficulty as to date there is no social support measure validated amongst CALD communities.

## Conclusions and recommendations for future research

The results of the current study have suggested that Iraqi Arabic speaking women experienced the support group intervention as socially acceptable and relevant as it incorporated their explanatory models of motherhood, migration, stress, and depression. The use of a group intervention that targets the whole community rather than individuals within that community appeared to provide an opportunity for education and support whilst decreasing the stigma surrounding their perceived inability to cope. Employing culturally sensitive facilitators who were able to build trust and confidence within the group and were prepared to work within the clients' explanatory models was important for the success of this intervention. In addition, the provision of practical components such as transport, childcare facilities and interpreters appeared to have been essential in facilitating attendance.

Future research needs to incorporate the use of bilingual workers from the same cultural background to procure referrals. It may also be appropriate to incorporate a session that addresses the spirituality aspects of this cultural group as this was referred to in group discussion. In addition, the incorporation of an analysis examining the effects of different facilitator's qualities independent of the program content may also be useful. There is also a need to use a more relevant measure is required to assess social support. A randomized controlled trial of the intervention is required to more systematically evaluate the efficacy of the program in terms of reducing PND symptoms and improving social support. Given the acceptability of this pilot intervention, further research is required to determine if these results are supported in other cultural contexts and with more members from similar cultural backgrounds. The current intervention is likely to be useful as a model for a culturally appropriate support group that can be used across CALD communities, resulting in a greater utilization of culturally appropriate services by CALD women.

### Conflict of interest statement

The authors declare that the research was conducted in the absence of any commercial or financial relationships that could be construed as a potential conflict of interest.
